# Physicochemical traits of Holstein loin and top round veal from two slaughter age groups

**DOI:** 10.1186/s40781-015-0058-0

**Published:** 2015-07-15

**Authors:** Dong-Gyun Yim, Sang-Woon Park, Ku-Young Chung

**Affiliations:** Department of Health Administration and Food Hygiene, Jinju Health College, Jinju, 660-757 South Korea; Department of Animal Science and Resources, Sangji University, Wonju, 220-702 South Korea

**Keywords:** Holstein veal, Loin, Top round, Meat quality, Slaughter age

## Abstract

The objective of this study was to investigate the physicochemical and microbial quality of loin (*m. longissimus dorsi*) and top round (*m. Semimembranosus*) in Holstein veal produced from two slaughter age groups (5 and 8 months of age). A total of 20 Holstein calves were randomly selected from a local cattle farm. The slaughtered cold carcasses were vacuum-packaged. The samples were analyzed for proximate composition and physicochemical analyses and stored for 1, 7, 10, 20 and 30 days for microbiological analyses. Fat and protein contents of loin for the 8 month group were higher than those for the 5 month groups (*p* < 0.05). For both loin and top round muscles, the pH, cooking loss and the shear force values for the 5 month group was higher than those for the 8 month group (*p* < 0.05). On the other hands, the water-holding capacity (WHC) for the 8 month group was higher than those for the 5 month group (*p* < 0.05). In terms of meat color, CIE L* (lightness) for both muscle were higher in the 5 month group than in the 8 month groups. On the other hands, a* (redness) were higher in the 8 month group than in the 5 month groups (*p* < 0.05). Total aerobic counts in all samples remained up to 30 days at values less than 7 log CFU/g. However, there was no significant difference for both muscles between the two age groups. The results indicate that Holstein muscles from the 8 month group had desirable quality properties than those from the 5 month group.

## Background

Holstein is the premier dairy breed with a high potential for milk production [1] and is spread all over South Korea. In Korea, Holstein cattle have introduced and been raised as a domestic stock since 1903 [2]. The statistics indicate that about 45,351 Holstein cow and 70,000 Holstein beef were slaughtered in 2012. The frequencies of quality grading above grade 1 for Holstein steers were only 9.0 % in 2013 [3]. Thus, Holstein beef have been not popular and utilized limitedly because it has inferior palatability characteristics as compared to Hanwoo. Some Holstein dairy farmers tried to produce the highly marbled Holstein steer beef using a longer feeding period, but this was not financially advantageous for them, due to the expensive feeding cost and low feeding efficiency.

Traditionally, veal has been of substantial value associated with a low fat content, and a good flavor to many countries [4]. According to Council Regulation (EC) No 361/2008 of April 14th [5], veal is described as the meat from unweaned calves that are slaughtered when they are no more than 8 months old. The European Commission differentiates veal as meat derived from calves of 16–19 week of age [6]. Currently, young Holstein bulls have been problematic for a livestock raiser in Korea. The farmers face serious challenges when they have new-born, male veal, given the unstable market price and low valuation of this product in the domestic beef market [7]. Therefore, the farmers found a solution to advance the slaughtering time by veal production. The average slaughtering times of Holstein steer were from 20 to 22 months. The slaughter ages in production of veal were between 5 and 8 months in most of countries [5]. Thus, it needs to compare the meat quality parameters between five and eight age groups. A number of publication have focused on meat quality of Hanwoo, but meat quality attributes of Holstein calves (bulls and steers) born and raised in Korea have rarely been assessed. Especially, very few publications have dealt with the effects of slaughter age on carcass and meat quality of young Holstein bulls. Therefore, the aim of this study was to compare the physicochemical and microbial quality characteristics of loin (*m. longissimus dorsi*) and top round (*m. Semimembranosus*) in Holstein veal produced from two slaughter age groups (5 and 8 months of age).

## Methods

### Animals and sample preparation

A total of 20 young Holstein bulls (5 and 8 months old) were randomly selected from a local cattle farm, South Korea. Experimental protocol was approved by the animal care committee of Sangji University, Republic of Korea. Ten calves at 5 months of age and 10 animals at 5 months of age were slaughtered and dressed in an officially approved slaughterhouse, according to standard methods, using a captive bolt stunner, followed by sticking and bleeding. The carcasses were immediately cooled at 0 °C for 24 h in a chilling room. The live and carcass weight for the 5 month group was average 159 and 72 kg, while those for the 8 month group was average 237 and 127 kg. Weight and percentage in primal cuts of Holstein veal calves from two slaughter age are presented in Table 1. Immediately after weighting, slices of loin (*Longissimus dorsi*) and top round (*Semimembranosus*) muscles were taken. After being vacuum packaged, the samples were transported to laboratory at university, South Korea. Immediately on arrival the samples were removed from vacuum packages. All subcutaneous fat and visible connective tissue of muscles were trimmed and re-vacuum packaged using vacuum package system (Vc999, K4N, Switzerland). Packaged samples were stored in refrigerator (CA-D17DC, LG, Korea) in which temperatures were controlled within 0 ± 1 °C of designated storage temperature. The samples for microbiological analyses were stored for 1, 7, 10, 20 and 30 days.

**Table 1:** Weight and percentage in primal cuts of Holstein veal from two slaughter age

	5 month		8 month	
	Weight (kg)	Percentage (%)	Weight (kg)	Percentage (%)
Tenderloin	1.3 ± 0.14	2.67	2.45 ± 0.64	2.80
Loin	5.30 ± 0.57	10.88	9.50 ± 2.97	10.86
Strip loin	1.3 ± 0.14	2.67	2.40 ± 1.27	2.74
Neck	3.55 ± 0.07	7.29	6.65 ± 1.77	7.60
Blade	5.25 ± 0.49	10.78	9.05 ± 2.05	10.34
Topside	5.75 ± 0.78	11.81	10.55 ± 2.62	12.06
Butt & rump	8.15 ± 0.92	16.74	13.95 ± 3.61	15.94
Brisket	5.00 ± 0.28	10.27	9.75 ± 3.89	11.14
Shank	4.45 ± 0.35	9.14	7.10 ± 1.41	8.11
Rib	8.65 ± 0.92	17.76	16.1 ± 7.64	18.40
Total	48.70	100	87.5	100

Values are Mean ± SD

### Proximate composition

Immediately after keeping in a chilling room, samples from each treatment were analyzed for proximate composition. All determinations were carried out on the homogenized samples, in triplicate. Moisture, fat, protein and ash were determined on samples using with a slightly modified method of AOAC [8].

### Physico-chemical analyses

The pH of samples was determined with a pH meter (PHM201, Radiometer, France). The pH values of samples were measured by blending a 10 g sample with 90 mL distilled water for 1 min in a homogenizer (Ultra-turrax, T25-S1, Germany). The water holding capacity (WHC) was conducted by a modification of the procedure of Grau and Hamm [9]. Briefly, a 300 mg sample of muscle was placed in a filter-press device and compressed for 2 min. WHC was calculated from duplicate samples as a ratio of the meat film area to the total area; hence, a larger value suggests a higher WHC. WHC(%) was calculated as follows: WHC (%) = 100- [total meat area/meat film area × 100]. For cooking loss, after the samples were thawed at 4 °C overnight before analyses and sliced with a thickness of 2 cm. The samples were weighed and cooked in an electric grill (EMG-533, AIJIA electric appliance, China) until they reached a final internal temperature of 70 °C. Cooking loss was determined by the ratio of the difference between raw weight and final cooked weight as follows: Cooking loss (%) = 100 × (raw weight - final cooked weight)/raw weight. Shear force values were measured by the method described by the procedure of Bourne [10]. The samples were prepared a cubic form (30 × 30 × 20 mm) and six cores of 1.27 cm in diameter were drilled parallel to the muscle fiber from each sample. Each core was sheared once with a Warner-Bratzler shear attachment using a texture analyzer (TA-XT2, Stable Micro System Ltd., U.K.). The maximum shear force value (kg) was recorded for each sample. Test and post-test speeds were set at 1.0 mm/s. Color measurements were taken using a Minolta chromameter (CR-410, Minolta Co. Ltd., Japan). CIE L*, a* and b* values were determined with measurements standardized with respect to a white calibration plate (L* = 94.4, a* = 0.313, b* = 0.319) after 30 min blooming at room temperature. Color measurements for each of three replicates, always trying to avoid area with excess fat were taken and the value was recorded.

### Microbiological analysis

Samples were subjected to microbiological analysis to monitor the dynamic changes in the populations responsible for the aging of the veal samples and their hygienic quality. The samples (10 g) were homogenized with 90 mL of 0.1 % sterile peptone water using a Stomacher Lab blender (Interscience BagMixers, Hanover, MA, USA) for 2 min and serially diluted with saline solution by 10-fold. Total aerobic plate counts were enumerated on plate count agar (Difco^TM^, Laboratories, Detroit, MI, USA) at 37 °C for 48 h. Bacterial counts were expressed as colony forming units per gram of sample (CFU/g).

### Statistical methods

The experiment had three replications. An analysis of variance (ANOVA) were performed on all the variables measured using the General Linear Model (GLM) procedure of the SAS statistical package [11]. The *t*-test (*p* < 0.05) was used to determine differences among the treatment means. Mean values and standard deviations were reported.

## Result and discussion

### Proximate composition

The proximate composition of loin (*m. longissimus dorsi*) and top round (*m. Semimembranosus*) of Holstein veal with two slaughter age is compared in Table 2. Fat and protein contents of the loin differed between two age groups (*p* < 0.05). Fat and protein contents for the 8 month group were higher than those for the 5 month groups. This is in similar to the ones found by Cho et al. [7] who indicated the protein and fat contents were increased with increasing month of age. Similarly, previous studies [12, 13] also found that protein content in beef loin muscles increased with advancing age. Tuma et al. [14] showed the *longissimus dorsi* muscle from the 6-month calves contained 72.63 % moisture, 21.24 % protein, 1.1 % ash content.

**Table 2:** Proximate composition of M. *longissimus dorsi* and *Semimembranosus* of Holstein veal with two slaughter age

	Month of age	Cut	
		*Longissimus dorsi*	*Semimembranosus*
Water (%)	5	76.46 ± 0.36	75.54 ± 0.64
	8	75.16 ± 0.36	76.16 ± 0.10
Fat (%)	5	0.55 ± 0.04^b^	0.90 ± 0.39
	8	1.52 ± 0.81^a^	0.42 ± 0.25
Protein (%)	5	20.93 ± 0.30^b^	21.36 ± 0.40
	8	22.54 ± 2.37^a^	21.84 ± 1.45
Ash (%)	5	1.09 ± 0.01	0.99 ± 0.16
	8	1.12 ± 0.08	1.26 ± 0.16

^a,b^means with different superscripts in the same column are significantly different (*P* < 0.05)

All values are mean ± standard deviation (n = 10)

### Physicochemical qualities

Physicochemical traits of loin (*m. longissimus dorsi*) and top round (*m. Semimembranosus*) of Holstein veal with two slaughter age were shown in Table 3. pH values of both muscles for the 5 month group were higher than those for the 8 month groups. These pH values are similar to those reported by authors [7, 14] have shown that pH values were decreased with advancing age. Previous study has reported pH value in veal could be correlated with meat color [15].

**Table 3:** Physicochemical traits of M. *longissimus dorsi* and *Semimembranosus* of Holstein veal with two slaughter age

	Month of age	Cut	
		*Longissimus dorsi*	*Semimembranosus*
pH	5	5.77 ± 0.26^a^	5.73 ± 0.10^a^
	8	5.31 ± 0.71^b^	5.21 ± 0.52^b^
WHC (%)	5	29.00 ± 1.58^b^	33.17 ± 2.84^b^
	8	44.81 ± 3.07^a^	47.96 ± 2.73^a^
Cooking loss (%)	5	41.07 ± 0.59^a^	42.12 ± 3.35^a^
	8	28.68 ± 0.35^b^	34.26 ± 1.12^b^
Shear force (kg)	5	13.26 ± 1.12^a^	10.40 ± 1.72^a^
	8	9.35 ± 1.50^b^	9.61 ± 0.04^b^
L*	5	50.44 ± 1.74^a^	49.67 ± 1.00^a^
	8	40.96 ± 2.56^b^	40.56 ± 0.44^b^
a*	5	10.21 ± 0.47^b^	12.15 ± 0.96^b^
	8	12.58 ± 0.48^a^	17.76 ± 0.92^a^
b*	5	6.41 ± 0.09	5.59 ± 1.69
	8	4.63 ± 3.14	3.87 ± 3.22

^a,b^means with different superscripts in the same column are significantly different (*P* < 0.05)

All values are mean ± standard deviation (n = 10)

As shown in Table 3, the water-holding capacity (WHC) of both muscles for the 8 month group was significantly higher than those for the 5 month group. This is in agreement with previous reports [7, 16] has indicated WHC in Holstein loin muscles increased with older age. On the other hands, Cooking loss and the shear force values of both muscles for the 5 month group was significantly higher than those for the 8 month group. Similar findings were obtained by authors [13] showing cooking loss decreased with increasing age. Generally, the beef muscle becomes tough with increasing age of the animal, indicating a possible structural change in collagen [12]. However, this is not in agreement with present result. In present study, the higher intramuscular fat for the 8 month group could be a crucial factor for the lower shear force values. Shear force values were negatively related to intramuscular fat content in numerous studies [17–19].

Basically, consumers are believed to assess veal quality on the lean color [6]. For meat color, CIE L* (lightness) of both muscles for the 5 month group were higher than those for the 8 month groups. On the other hands, a* (redness) of both muscles for the 8 month group were higher than those for the 5 month groups (Table 3). This is demonstrated by the findings [13] that L* decreased with older age, whereas a* increased. Similarly, Tuma et al. [20] also showed longissimus dorsi steaks were darker red with increasing month of age. Low L* values may be attributed to increased myoglobin and decreased muscle glycogen [21]. Muscle color varies, and anatomical location of the muscle influences most color traits, including pigment content, reflectance, redness, and the rate of meat discoloration [7]. Color was also correlated with the ultimate pH, such that lightness, redness, and reflectance decreased with an increase in the ultimate pH [15].

### Microbiological analyses

**Fig. 1:**
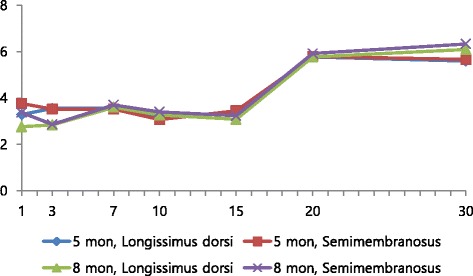
Changes of total plate counts Holstein veal with two slaughter age during storage. All values are mean ± standard deviation (n = 10)

Changes of microbial populations of loin (*m. longissimus dorsi*) and top round (*m. Semimembranosus*) of Holstein veal with two slaughter age during the 30 day storage period were shown in Fig. 1. The population of total aerobic increased slowly regardless of month of age during storage. However, there was no significant difference for loin and top round muscle between the two slaughter age groups. The samples remained below the microbiological guidelines for meat maximum limit (below 7 log CFU/g) [22] up until 30 days. In reviewing the literature, vacuum packaging retards microbiological growth, and delays the development of spoilage due to slow proliferation of bacteria capable of tolerating anaerobic conditions [23]. Maximum bacterial numbers are reached after 5 weeks of vacuum packaged storage [24]. The bacterial counts of 7 log CFU/g is the approximate point at which meat would be considered to be spoiled or unacceptable [25]. The maximum acceptable counts for packed meat, not matured, are below 10^7^ for total counts as recommended [22]. In the present work, vacuum-packaged beefs during the cold storage period for 30 days remained within the acceptable limits established by Korea MFDS. Therefore, the shelf-life of Holstein veal samples stored at 0 °C under vacuum conditions would be within 30 days. Bacteria counts of Holstein veal appeared to be not related to month of age in this study.

## Conclusion

Slaughter age affect the proximate composition and physicochemical traits of Holstein veal. The results indicate that the muscles from the 8 month group in Holstein calf had desirable quality properties when compared to the 5 month group. The results of this study will give objective information on the meat quality depending on different age of young Holstein bulls for consumers. Further research should be done to find a better Holstein veal quality in the aspects of functional, sensory, economic and health benefits. The advantages of savings in feed cost should be considered for Holstein farmers, by advancing existing slaughtering age from 20 month to less than 8 month of age. Therefore, the production of Holstein calf beef could contribute to discrimination of Holstein beef from Hanwoo and imported beef in the domestic beef market.
